# More tools to uncover cancer cells

**DOI:** 10.1016/j.omton.2026.201136

**Published:** 2026-02-04

**Authors:** María Florencia Pignataro, Javier Santos

**Affiliations:** 1 Instituto de Biociencias, Biotecnología y Biología Traslacional (iB3), Universidad de Buenos Aires, Buenos Aires, Argentina; 2 Departamento de Fisiología, Biología Molecular y Celular (DFBMC), Facultad de Ciencias Exactas y Naturales (FCEN), Universidad de Buenos Aires (UBA), Buenos Aires, Argentina; 3 Consejo Nacional de Investigaciones Científicas y Técnicas (CONICET), Godoy Cruz 2290 (C1425FQB) CABA, Buenos Aires, Argentina

## Tumor-associated antigens and tumor-specific antigens

Tumor-associated antigens (TAAs)—proteins often overexpressed in cancer cells—represent a landmark discovery in cancer immunology. The immune system’s ability to target TAAs is crucial for early cancer detection (via biomarkers), assessing disease progression, determining prognosis, and high-accuracy monitoring. TAAs are essential in cancer immunotherapy. A seminal contribution in this field was made by van der Bruggen and colleagues, with “MAGE” (melanoma antigen).^1^

In a recent study, the team led by Frédéric Pecorari and Christophe Blanquart focused on human mesothelin (hMSLN)^2^ as a target for binder selection and the design of new, affordable tools. The glycoprotein hMSLN is a TAA that is overexpressed in many solid tumors and a target for therapeutic development.^3^ The authors developed new malleable molecular tools based on Affitin selection through ribosome display. Notably, they combined a bifunctional Affitin with a nanobody to simultaneously bind hMSLN and CD16, thereby functioning as a natural killer cell engager (BiKE).^4^^,^^5^

## Affitins and nanobodies as scaffolds

**Figure 1:**
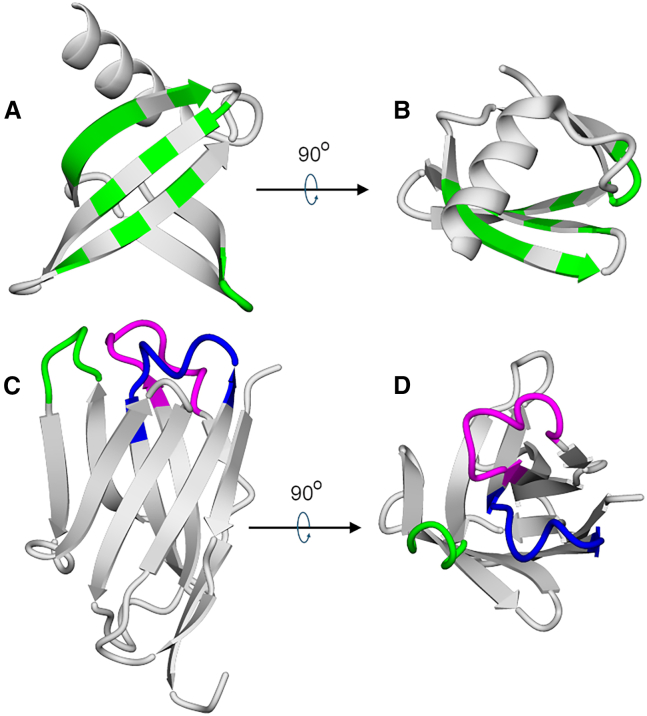
Affitins and nanobodies as scaffolds (A and B) Two views of an Affitin (Sac7d) showing a flat platform of binding with 10–14 residues in green that are randomized in the Affitin DNA library (positions 8, 9, 10, 11, 21, 22, 23, 24, 26, 29, 31, 33, 40, 42, and 44), resulting in a highly diverse collection of protein sequences. (C and D) Two views of a nanobody (a V_HH_ domain). CDR1, CDR2, and CDR3 are shown in blue, green, and magenta, respectively. CDR3 may be considerably longer than CDR1 and CDR2, and this fact confers specific binding properties on the NB.

Engineering specific and high-affinity binding to TAAs and tumor-specific antigens (TSAs) is a challenging task that organisms tackle by employing very large scaffolds: antibodies. Conversely, in the laboratory, different strategies have been employed. Frédéric Pecorari and coworkers previously showed that a small scaffold (66 amino acids, 7 kDa, Figures 1A and 1B), Sac7d, an archaeal DNA-binding protein, can be effectively used for molecular recognition.^6^ To do this, 10 residues on the surface of Sac7d are fully randomized to create a DNA library with 10^12^ different Sac7d sequences. From this large library, the binders that recognize a specific target protein are selected through several cycles of ribosome display.^7^ In the ribosome display selection process, each mRNA remains linked to a ribosome and a nascent binder encoded by this mRNA, initiating a “selection journey.”

The synthetic binders derived from this process were called Affitins. It is important to note that, generally, Affitins have a well-defined, highly stable tertiary structure, formed by a beta-barrel capped with a C-terminal helix, and lack a disulfide bridge. In fact, Sac7d remains stable at extremely low pH values and high temperatures, such as 90°C. The selected binders typically have dissociation constants in the nanomolar range. One crucial aspect is that Affitins can be easily produced in *E. coli*. Therefore, Affitins offer a versatile method for developing customized affinity tools for challenging applications. This astounding idea laid the foundation for subsequent developments and is recognized as one of the most significant steps in synthetic binder development.

On the other hand, the presence of heavy-chain-only antibodies in camelids and sharks has opened new opportunities and applications in protein engineering. Each chain of this type of antibody contains a 15 kDa domain (V_HH_) that comprises three complementarity-determining regions (CDRs) that directly interact with the target. This domain was named nanobody (NB, Figures 1B and 1C).^8^

Typically, after a camelid immunization with a protein target, heavy-chain-only antibodies, which have evolved to act as high-affinity binders without a light chain, can be produced, along with a library of V_HH_ sequences. The specific NBs against the target can then be further selected using phage display.^9^ Alternatively, specific V_HH_ domains can be obtained directly from large synthetic DNA libraries via *in vitro* selection using phage or ribosome display strategies.^10^ Remarkably, the potential of V_HH_ domains was early envisioned. Although NBs were proposed as promising tools for cancer treatment over 20 years ago, the concept gained momentum due to their broad potential and wide range of applications in this field.^10^ NBs are soluble, highly stable proteins with high Tm values, resistance to aggregation, and temperature-induced unfolding. Because NBs are smaller than traditional monoclonal antibodies, NBs exhibit better tissue penetration. Bifunctional NBs can be created by combining two V_HH_ domains with a flexible linker, enabling simultaneous binding to different targets or to multiple copies of the same target, thereby increasing avidity. NBs can carry drugs, stabilize intrinsically unstable proteins, and be expressed intracellularly in different subcellular compartments.

## The development of a potent Affitin-based tool for the therapy of MSLN-expressing cancers

**Figure 2:**
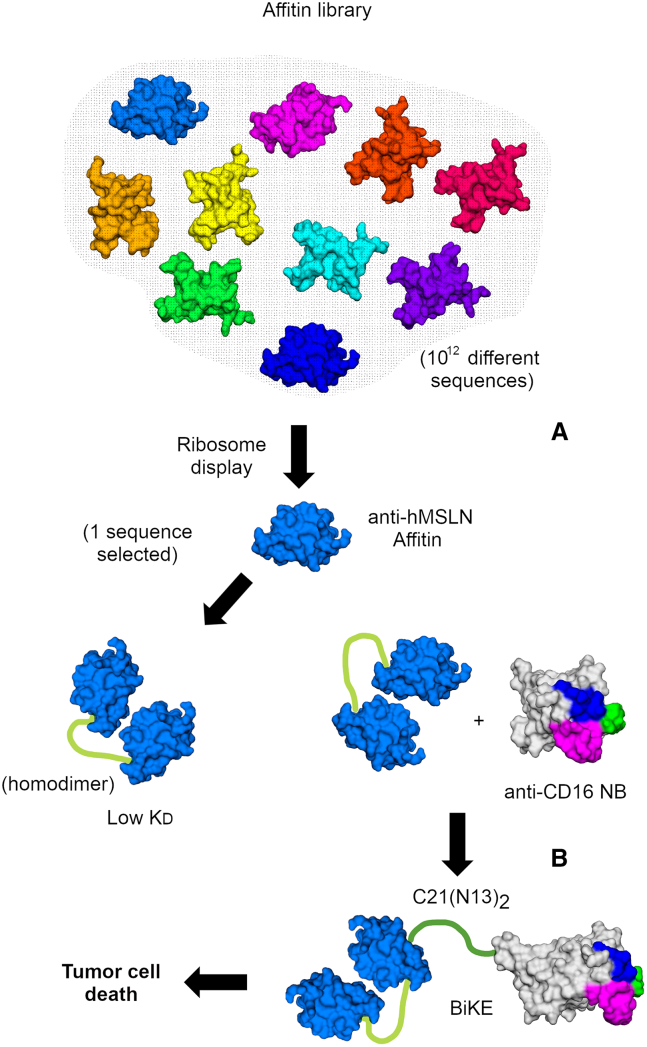
Building a BiKE (A) A randomized Affitin DNA library undergoes multiple rounds of ribosome display, with selection rounds alternating between using the full-length recombinant hMSLN or a recombinant fragment as the target, and an elution-by-competition setup. Anti-hMSLN affinities are selected and sequenced. One of these proves to be an effective binder (Affitin N13). Homodimerization through linker insertion results in a higher affinity homodimer (lower observed K_D_). (B) The homodimer is then linked to an anti-CD16 NB to activate natural killer cells against hMSLN-expressing cancer cells, leading to tumor cell death.

Using several rounds of ribosome display and elution by competition, the authors selected Affitins that bind to the same site as a monoclonal antibody currently evaluated in clinical trials (Figure 2A). Thus, the authors were able to target a precise epitope. Remarkably, they studied 27 Affitin candidates, demonstrating a substantial volume of work. Two Affitins (N13 and N18) exhibited *K*_D_ values of 35 nM. While N13 was very stable, N18 exhibited a more complex behavior, with several unfolding transitions and aggregation, presumably reflecting an intrinsic tendency to form dimers. On the other hand, the Affitins exhibit key malleability that is essential for protein engineering. Affitins were biotinylated, and binding to Meso34-MSLN cell lines was monitored by flow cytometry after tetramerization on streptavidin labeled with the Alexa Fluor 647 fluorophore. Given the limitations of streptavidin-based tools for human procedures and to enhance the affinity of the Affitins in the cellular environment, a stable dimeric form of the Affitin N13 was developed, demonstrating a significantly lower *K*_D_ (0.57 nM) and binding to the cancer cell surface, and a low-immunogenic linker, critical for use in humans, was included. As expected, the *K*_D_ kinetic coefficient was reduced by more than 80-fold. To evaluate the therapeutic potential of Affitin N13*,* the authors developed bispecific BiKEs,^4^^,^^5^ including either a monomer or a homodimer of the Affitin N13 fused to the C21 anti-CD16 NB, C21(N13) and C21(N13)_2_, respectively (Figure 2B). Remarkably, the dimerization of N13 also greatly improved the performance of the BiKE, as natural killer (NK)-mediated cytotoxicity was higher with the C21(N13)_2_ BiKE than with C21(N13).

Selecting or designing high-affinity binders (with equilibrium dissociation constants in the low-nanomolar to sub-nanomolar range) remains a challenging goal. However, new strategies, including the use of artificial intelligence tools, could overcome obstacles and can be usefully combined with phage display, yeast display, or even cell-free selection tools. The future is promising: a fine-tuned RFdiffusion combined with yeast display screening has enabled the *de novo* generation of specific V_HH_ domains that not only exhibit nanomolar and sub-nanomolar K_D_ but also accurately target specific binding epitopes.^11^ If this reflects the current state of the art, we wonder whether there is a limit to what this synergism can achieve.

## Declaration of interests

The authors declare no competing interests.
